# Injury-related deaths before and during the Islamic State insurgency – Baghdad, Iraq, 2010–2015

**DOI:** 10.1186/s13031-020-0252-7

**Published:** 2020-02-18

**Authors:** Matthew Goers, Eva Leidman, Abdul-Salam Saleh Sultran, Ahmed Hassan, Oleg Bilukha

**Affiliations:** 1 Centers for Disease Control and Prevention, Center for Global Health, Division of Global Health Protection, Atlanta, GA USA; 2 National Training and Human Development Center, Iraq Ministry of Health, Bab Al Mu’adham Street, Baghdad, Iraq; 3 Medical Operations and Specialized Services Directorate, Operations Department, Iraq Ministry of Health, Al Adham Street, Baghdad, Iraq

**Keywords:** Iraq, Conflict, Injury mortality, Islamic state, Gunfire, Explosions, Assaults, Interpersonal violence, Traffic accidents, Unintentional injuries

## Abstract

**Background:**

Following a period of low intensity conflict during 2009–2012, the emergence of the Islamic State of Iraq and Levant (or Islamic State) in 2013 was associated with a resurgence of violence in Baghdad, Iraq’s capital and largest city. We evaluated trends in injury-related deaths in Baghdad before and during the Islamic State insurgency.

**Methods:**

Iraqi National Injury Mortality Surveillance System prospectively collects information on fatal injuries from governorate coroner offices using standardized reporting forms. Trained coroner clerks collect information on victim demographics, intention of injury and mechanism of injury during medical examinations using reports from police and families. We analyzed data on all deaths reported by the Baghdad Forensic Institute from January 1, 2010 to December 31, 2015.

**Results:**

There were 17,555 injury-related deaths with documented intent and mechanism (range 2385–3347 per year): 6241 from gunfire (36%), 1381 explosions (8%), 1348 non-gunfire assaults (8%), 3435 traffic accidents (20%), and 5150 other unintentional injuries (29%). Rates of gunfire (23.45 per 100,000) and explosion (5.90 per 100,000) deaths were significantly higher in 2014 than in all other years during the review period (*p* < 0.001 and *p* = 0.03, respectively). During the same period from 2010 to 2015, traffic accident deaths declined significantly from 13.29 to 6.35 (*p* = 0.002), with declines observed primarily among pedestrians. Trends in the rate of non-gunfire-assault and unintentional deaths, comparing 2010 and 2015, were not significant (*p* = 0.12 and *p* = 0.63, respectively). Unintentional deaths were mostly attributed to burns (46%) and electricity-related injuries (31%). The proportion of both females and children was highest in unintentional injury deaths.

**Conclusions:**

During the study period, deaths from both gunfire and explosions in Baghdad peaked in 2014, corresponding with emergence of the Islamic State. Trends suggest a potential impact of insurgency-related activity on other injuries as evidenced by a decrease in the death rate from traffic accidents. The decreased traffic-related death rate could be from decreased vehicle and pedestrian activity during times of violence. Monitoring trends in injury mortality during conflict allows Iraq to identify priority injury causes to inform public health interventions.

## Background

Since the United States (US) led invasion in 2003, Iraq has experienced intermittent, high intensity conflict. Multiple sources, including retrospective surveys and prospective media-based surveillance, demonstrate similar trends of conflict violence in Iraq. While these sources differ on the exact number of civilian deaths, all show that conflict-related deaths increased from 2003 to around 2007 then decreased in 2008, corresponding with increased US troop presence [1]. This decrease continued through the US military withdrawal in 2010 with relatively low levels of conflict-related deaths through 2012 [2–11].

In 2013, the emergence of the Islamic State of Iraq and Levant (also referred to as the Islamic State, ISIL, ISIS or Daesh) led to renewed conflict throughout Iraq. In April 2013, the Islamic State began its heaviest offensives, leading to significant territorial gains in northwest Iraq [12]. This renewed conflict resulted in levels of civilian casualties nationally higher than at any point since 2008 [13]. Baghdad, Iraq’s capital and largest city, shared a large burden of this violence. The US Department of State reported that 41% of all terror attacks in Iraq in 2015 occurred in Baghdad [14, 15].

In 2010, Iraq established its first injury mortality surveillance system with assistance from World Health Organization (WHO) and the US Centers for Disease Control and Prevention (CDC). With this system in place, Iraq has been able to systematically collect data on both conflict and non-conflict injury mortality. Data have been collected during periods of both relatively low levels of conflict as well as higher levels of conflict, including the period following the Islamic State’s emergence in 2013. It is uncommon for a country to have a functioning injury mortality surveillance system during times of conflict [16]. In 2010 when Iraq established its system, only 83 countries had mortality surveillance systems, of which only 20 were considered high quality [17]. However, most of the quality systems were in high-income countries and none were affected by conflict. Thus, the systematic collection of data from Iraq’s surveillance system provides a rare opportunity to describe the trends and magnitude of injury mortality in a conflict-affected country.

To evaluate trends before and after the emergence of the Islamic State, we analyzed surveillance data from Baghdad governorate. Baghdad is Iraq’s most populous governorate, and one of the few governorates heavily impacted by insurgency activity with consistent surveillance data from 2010 to 2015 [12, 14]. The objective of this analysis was to provide a description of conflict and non-conflict injury deaths as well as annual injury mortality trends in Baghdad, before and after the emergence of the Islamic State.

## Methods

Data on all injury-related deaths from January 1, 2010 to December 31, 2015 were obtained from Iraq’s Injury Mortality Surveillance System. The surveillance system is operated by Iraqi Ministry of Health with technical support from WHO and CDC, and includes data on all deaths from injury reported to governorate coroner offices. Coroners are responsible for and legally required to issue death certificates for all injury-related deaths in Iraq [18]. The analysis presented here includes data on all deaths from injury reported to the Baghdad Forensic Institute, the coroner office serving Baghdad governorate.

The case definition used by the surveillance system included all persons who died because of an external injury. Fatalities that occurred in police custody were included. However, fatalities from legal interventions (e.g., actions by police) were not included. Deaths from interpersonal violence in the context of sexual assault were classified based on the primary mechanism of injury, without indication of sexual assault as a cause.

Trained clerks in coroner offices collected information on injury deaths on a standardized reporting form. The forms were completed based on all available information including physical examinations and reports from police and family members. Standardized forms collected information on governorate of incident, victim demographics, death certificate number, date of arrival to coroner office, date of death certificate issue, date of injury, circumstances of the incident, mechanism of injury and intention of injury. For the presented analysis, if date of death certification was unavailable, date of cadaver discovery was used instead.

During analysis, persons less than 18 years old were classified as children. Injury deaths were reclassified into five intent/mechanism categories, including three intentional injury categories (deaths from gunfire, explosions, and non-gunfire-assault [e.g., blunt or sharp weapon injuries]) and two unintentional categories (deaths from traffic accident injuries and other unintentional injuries [e.g., burns, falls, electrical injuries]). The phrase “conflict-related” is used when referring to both gunfire and explosion deaths. All traffic accident deaths were classified as unintentional without consideration of reported intent. Traffic accident deaths involving either drivers or passengers of motor vehicles (e.g., car, motorcycle) were classified as vehicle occupant deaths. Due to concerns for underreporting, deaths from self-harm were excluded from analysis. Rates were calculated using population estimates from the World Bank. The World Bank figures are United Nations World Urbanization Prospects modeled estimations for the metropolitan area of Baghdad. The modeled figures are adjusted for an estimated annual growth rate of 2.4% per year, but do not reflect the notable population displacement impacting Baghdad during the study period [19].

Trends in the number of fatalities each year, overall and by age and sex were calculated and examined. Changes in annual rates of death for all categories, as well as differences in the rate of deaths in pre-insurgency (2010–2012) and post-insurgency (2013–2015) periods, were compared using a t-test assuming unequal variance by comparing the monthly means during each period. For gunfire and explosion-related fatality rates, we also tested for differences comparing 2014 with all other years. Confidence intervals were calculated based on procedures recommended by the National Center for Health Statistics [20]. Trends were visualized using local weighted regression (loess).

We checked the database for duplicate entries by comparing victim demographics, the time and location of incident, and mechanism of injury. Data were entered in Epi Info™ and statistical analysis was performed using SAS™ statistical software (version 9.4) [21, 22]. Figures of monthly and annual trends were generated in R studio (version 3.4.1) [23]. The Institutional Review Board of US CDC determined this study to be “non-research” as it entailed secondary analysis of routinely collected public health surveillance data from 2010 to 2015. Personal identifiers were not included in the final dataset used for analysis.

## Results

In total, 17,848 injury fatalities were reported by the Baghdad Forensic Institute from 2010 to 2015. Of those, 293 (2%) had missing data on intention or mechanism of injury and were removed from analysis. Of the remaining 17,555 deaths with documented intent and mechanism, 3070 (17%) were missing information on age and 212 (1%) missing information on sex. The proportion of those with missing age and sex was highest in conflict-related deaths. Those with missing information on sex or age were included in the overall analysis, but excluded from sub-analyses based on sex or age, respectively. Overall, 6241 (36%) deaths were from gunfire, 1381 from explosions (8%), 1348 from non-gunfire-assault (8%), 3435 from traffic accidents (20%) and 5150 from other unintentional injuries (29%).

Of those with documented age (*n* = 14,485), 25% were children (Table 1). For those with documented sex (*n* = 17,343), 27% were female. The highest percentages of children and females were among deaths from other unintentional injuries (38% children, 47% females). The lowest proportion of children and females were in deaths due to conflict; children represented 11% of gunfire deaths and 17% of explosion deaths, and females represented 16% of gunfire deaths and 10% of explosion deaths.

**Table 1:** Annual Injury-Related Deaths by Category, Sex and Age — Baghdad, Iraq, 2010–2015

Year		2010	2011	2012	2013	2014	2015	Total
Explosions^a^	N (%)	276 (10%)	193 (8%)	156 (6%)	222 (7%)	366 (11%)	168 (6%)	1381 (8%)
	Rate per 100,000 persons (CI)	4.88 (4.32–5.49)	3.34 (2.88–3.84)	2.63 (2.24–3.08)	3.66 (3.2–4.18)	5.90 (5.31–6.53)	2.65 (2.26–3.08)	
Female^b^	n/N (%)	28/248 (11%)	15/181 (8%)	19/142 (13%)	13/200 (7%)	28/332 (8%)	24/151 (16%)	127/1254 (10%)
Children^c^	n/N (%)	26/189 (14%)	20/141 (14%)	19/111 (17%)	24/136 (18%)	34/210 (16%)	35/121 (29%)	158/908 (17%)
Gunfire^a^	N (%)	823 (29%)	736 (26%)	806 (28%)	1368 (48%)	1455 (51%)	1053 (37%)	6241 (36%)
	Rate per 100,000 persons (CI)	14.56 (13.58–15.59)	12.72 (11.82–13.68)	13.61 (12.69–14.58)	22.57 (21.39–23.80)	23.45 (22.26–24.59)	16.58 (15.59–17.61)	
Female^b^	n/N (%)	131/816 (16%)	122/733 (17%)	141/801 (18%)	211/1361 (16%)	214/1446 (15%)	147/1022 (14%)	966/6179 (16%)
Children^c^	n/N (%)	74/657 (11%)	64/618 (10%)	70/656 (11%)	88/1047 (8%)	103/1026 (10%)	143/807 (18%)	542/4811 (11%)
Non-Gunfire Assaults^a^	N (%)	187 (7%)	158 (7%)	184 (7%)	231 (7%)	252 (8%)	336 (12%)	1348 (8%)
	Rate per 100,000 persons (CI)	3.31 (2.85–3.82)	2.73 (2.32–3.19)	3.11 (2.67–3.59)	3.81 (3.34–4.34)	4.06 (3.58–4.59)	5.29 (4.74–5.89)	
Female^b^	n/N (%)	55/185 (30%)	43/158 (27%)	59/180 (33%)	61/225 (27%)	58/251 (23%)	114/333 (34%)	390/1332 (29%)
Children^c^	n/N (%)	25/127 (20%)	28/118 (24%)	33/145 (23%)	24/168 (14%)	37/181 (20%)	87/289 (30%)	234/1028 (23%)
Traffic Accidents^a^	N (%)	751 (27%)	617 (22%)	710 (25%)	396 (14%)	558 (20%)	403 (14%)	3435 (20%)
	Rate per 100,000 persons (CI)	13.29 (12.35–14.27)	10.67 (9.84–11.54)	11.99 (11.12–12.90)	6.53 (5.91–7.21)	8.99 (8.26–9.77)	6.35 (5.74–7.00)	
Female^b^	n/N (%)	162/751 (22%)	134/616 (22%)	173/708 (24%)	92/396 (23%)	142/557 (26%)	94/403 (23%)	797/3431 (23%)
Children^c^	n/N (%)	206/650 (32%)	172/541 (32%)	202/621 (33%)	101/372 (27%)	160/501 (32%)	107/365 (29%)	948/3050 (31%)
Other Unintentional Injuries^a^	N (%)	793 (28%)	681 (29%)	894 (33%)	1130 (34%)	710 (21%)	942 (32%)	5150 (29%)
	Rate per 100,000 persons (CI)	14.03 (13.07–15.04)	11.77 (10.90–12.69)	15.10 (14.12–16.12)	18.64 (17.57–19.76)	11.44 (10.62–12.32)	14.83 (13.90–15.81)	
Female^b^	n/N (%)	324/786 (41%)	332/668 (50%)	435/861 (51%)	506/1089 (47%)	326/707 (46%)	438/911 (48%)	2361/5022 (47%)
Children^c^	n/N (%)	275/685 (40%)	191/608 (31%)	283/773 (37%)	381/996 (38%)	277/662 (42%)	314/855 (37%)	1721/4579 (38%)
Total^a^	N	2830	2385	2750	3347	3341	2902	17,555
	Rate per 100,000 persons (CI)	50.07 (48.25–51.95)	41.23 (39.59–42.91)	46.44 (44.72–48.21)	55.22 (53.36–57.12)	53.85 (52.03–55.70)	45.69 (44.04–47.38)	
Female^b^	n/N (%)	700/2786 (25%)	646/2356 (27%)	827/2692 (31%)	883/3271 (27%)	768/3293 (23%)	817/2820 (29%)	4641/17218 (27%)
Children^c^	n/N (%)	606/2308 (26%)	475/2066 (23%)	607/2306 (26%)	618/2719 (23%)	611/2580 (24%)	686/2437 (28%)	3603/14416 (25%)

^a^ The number and rate presented are that of the specified mechanism/intention category among all injury-related deaths irrespective of documented age or sex data. Cell percentages represent the percent of total injury-related deaths for the given year (for annual columns) or for all years, 2010 through 2015 (for the total column)

^b^The number and percentage of females among all injury-related deaths from the specified mechanism/intention injury category. Cell percentages represent the percent of females among the mechanism-specific deaths for the given year (for annual columns) or for all years, 2010 through 2015 (for the total column). Figures exclude deaths without documented sex

^c^ The number and percentage of children (persons under 18 years of age) among all injury-related deaths from the specified mechanism/intention injury category. Cell percentages represent the percent of children among the mechanism-specific deaths for the given year (for annual columns) or for all years, 2010 through 2015 (for the total column). Figures exclude deaths without documented age

**Fig. 1:**
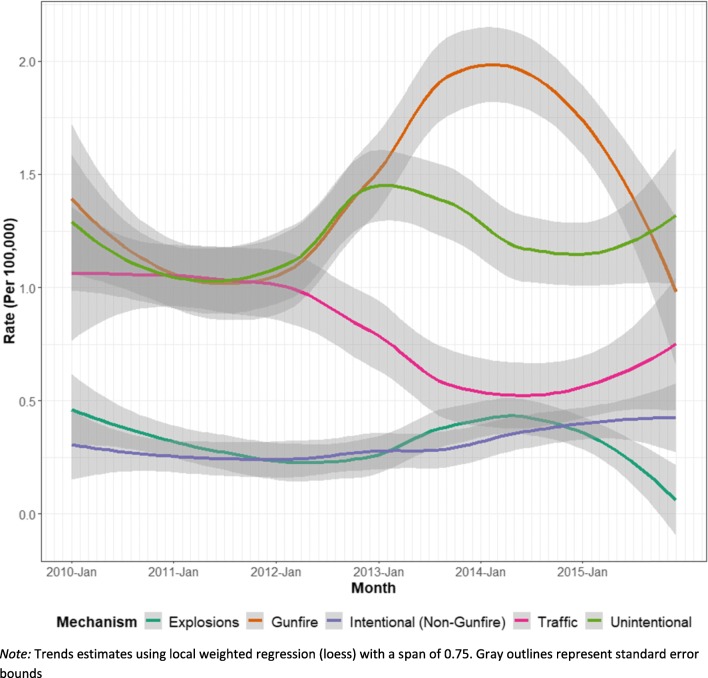
Monthly Injury-Related Deaths by Category — Baghdad, Iraq, 2010–2015 (*N* = 17,555)

Between 2010 and 2012 and 2013–2015 a significant increase was noted in the counts and rates of gunfire (*p* < 0.001), non-gunfire-related assault (*p* = 0.010), and total deaths (*p* = 0.02). The annual numbers and rates of fatal injuries, overall and in each injury category, were relatively stable from 2010 to 2012 (Table 1, Fig. 1). In 2013, however, notable changes were observed in each category. The rate for total injury-related deaths increased by 19% from 2012 to 2013 (*p* = 0.03). Rate of conflict-related deaths increased by 62% from 2012 to 2013 (*p* = 0.002). These increases continued in 2014, leading to a peak in conflict-related deaths for the study period. From 2010 to this peak in 2014, the rate of gunfire deaths increased by 61% (*p* < 0.001); the rate in 2014 was significantly higher than all other years during the review (p < 0.001). In 2010, conflict-related deaths accounted for 39% of total injury mortality, while in 2014 they accounted for 62% of total injury mortality. The annual rate of non-gunfire-assault increased by 60% between 2010 and 2015, however the increase was not significant (*p* = 0.12) (Fig. 1). The rate of explosion deaths increased between 2012 and 2014 and then declined in 2015, such that no significant increase was noted comparing the pre- and post- insurgency periods (*p* = 0.44). However, the monthly rates of explosion-related fatalities in 2014 were significantly higher than during the remainder of the study period (*p* = 0.03). Conversely, the rate of traffic accident deaths declined significantly (*p* < 0.001) from 2010–2012 to 2013–2015. Between 2010 and 2015 the rate of traffic accidents declined from 13.3 to 6.4 per 100,000 per year, a 52% decline (*p* = 0.002).

Tables 2, 3 and 4 present the counts and rates for primary mechanisms of injury for non-conflict injuries. The mechanisms of injury for deaths categorized as non-gunfire-assault are presented in Table 2. The majority of these deaths were from sharp weapon injuries (33%), blunt weapon injuries (26%) or assault-related suffocations (18%). No specific trend over the study period was noted in these subcategories (Table 2). The proportion of females was highest among assault-related suffocations deaths (44%) and assault-related burn deaths (42%). The proportion of children was highest for assault-related suffocations (37%). From 2010 to 2015, the number of female non-gunfire-related assault deaths rose by 107% and children deaths rose by 248%.

**Table 2:** Annual Non-Gunfire Assault Deaths by Injury Type, Sex and Age — Baghdad, Iraq, 2010–2015

Year		2010	2011	2012	2013	2014	2015	Total
Sharp Weapon^a^	N (%)	71 (39%)	75 (48%)	96 (53%)	66 (31%)	59 (24%)	64 (20%)	431 (33%)
	Rate per 100,000 persons (CI)	1.26 (0.98–1.56)	1.30 (1.02–1.61)	1.62 (1.31–1.96)	1.09 (0.84–1.37)	0.95 (0.72–1.21)	1.01 (0.78–1.27)	
Female^b^	n/N (%)	19/70 (27%)	18/74 (24%)	28/95 (30%)	16/64 (25%)	14/44 (32%)	20/61 (33%)	115/408 (28%)
Children^c^	n/N (%)	14/54 (26%)	11/61 (18%)	14/88 (16%)	4/57 (7%)	4/44 (9%)	15/60 (25%)	62/364 (17%)
Blunt Weapon^a^	N (%)	31 (17%)	26 (17%)	17 (9%)	70 (33%)	49 (20%)	144 (44%)	337 (26%)
	Rate per 100,000 persons (CI)	0.55 (0.37–0.76)	0.45 (0.29–0.64)	0.29 (0.17–0.44)	1.15 (0.90–1.44)	0.79 (0.58–1.03)	2.27 (1.91–2.67)	
Female^b^	n/N (%)	6/31 (19%)	2/25 (8%)	2/15 (13%)	11/69 (16%)	5/49 (10%)	33/143 (23%)	59/332 (18%)
Children^c^	n/N (%)	3/21 (14%)	2/17 (12%)	1/10 (10%)	5/56 (9%)	8/36 (22%)	40/125 (32%)	59/265 (22%)
Suffocation^a^	N (%)	1 (1%)	35 (22%)	65 (36%)	54 (25%)	59 (24%)	64 (20%)	228 (18%)
	Rate per 100,000 persons (CI)	0.02 (0.0–0.07)	0.60 (0.42–0.82)	1.10 (0.85–1.38)	0.89 (0.67–1.14)	0.95 (0.72–1.21)	1.01 (0.78–1.27)	
Female^b^	n/N (%)	1/1 (100%)	11/35 (31%)	28/63 (44%)	25/54 (46%)	4/10 (40%)	30/63 (48%)	99/226 (44%)
Children^c^	n/N (%)	0/0 (0%)	8/23 (35%)	17/43 (40%)	8/33 (24%)	4/10 (40%)	21/50 (42%)	58/159 (37%)
Burns^a^	N (%)	6 (3%)	8 (5%)	4 (2%)	9 (4%)	26 (11%)	34 (10%)	87 (7%)
	Rate per 100,000 persons (CI)	0.11 (0.04–0.21)	0.14 (0.06–0.25)	0.07 (0.02–0.15)	0.15 (0.07–0.26)	0.42 (0.27–0.59)	0.54 (0.37–0.73)	
Female^b^	n/N (%)	2/6 (33%)	6/8 (75%)	1/4 (25%)	2/6 (33%)	4/26 (15%)	19/32 (59%)	34/82 (42%)
Children^c^	n/N (%)	1/4 (25%)	4/7 (57%)	0/4 (0%)	0/3 (0%)	2/12 (17%)	4/31 (13%)	11/61 (18%)
Other^a^	N (%)	78 (42%)	14 (9%)	2 (1%)	32 (14%)	108 (43%)	31 (9%)	265 (20%)
	Rate per 100,000 persons (CI)	1.38 (1.09–1.70)	0.24 (0.13–0.38)	0.03 (0.0–0.09)	0.53 (0.36–0.73)	1.74 (1.43–2.21)	0.49 (0.33–0.67)	
Female^b^	n/N (%)	27/77 (35%)	6/14 (43%)	0/1 (0%)	7/30 (23%)	31/107 (29%)	12/31 (39%)	83/260 (32%)
Children^c^	n/N (%)	7/47 (15%)	6/9 (67%)	1/2 (50%)	7/22 (32%)	19/79 (24%)	7/24 (29%)	44/183 (24%)
Total^a^	N	187	158	184	231	252	336	1348
	Rate per 100,000 persons (CI)	3.31 (2.85–3.82)	2.73 (2.32–3.19)	3.11 (2.67–3.59)	3.81 (3.34–4.34)	4.06 (3.58–4.59)	5.29 (4.74–5.89)	

^a^ The number and rate presented are that of the specified sub category among all non-gunfire-assault deaths irrespective of documented age or sex data. Cell percentages represent the percent of non-gunfire-assault deaths for the given year (for annual columns) or for all years, 2010 through 2015 (for the total column)

^b^The number and percentage of females among all non-gunfire-assault deaths from the specified sub category. Cell percentages represent the percent of females among the sub category deaths for the given year (for annual columns) or for all years, 2010 through 2015 (for the total column). Figures exclude deaths without documented sex

^c^The number and percentage of children (persons under 18 years of age) among all non-gunfire-assault deaths from the specified sub category. Cell percentages represent the percent of children among the sub category deaths for the given year (for annual columns) or for all years, 2010 through 2015 (for the total column). Figures exclude deaths without documented age

**Table 3:** Annual Traffic Accident Deaths by Injury Type, Sex and Age — Baghdad, Iraq, 2010–2015

Year		2010	2011	2012	2013	2014	2015	Total
Pedestrian^a^	N (%)	546 (73%)	511 (83%)	674 (95%)	359 (91%)	427 (77%)	184 (46%)	2701 (79%)
	Rate per 100,000 persons (CI)	9.66 (8.87–10.51)	8.83 (8.08–9.63)	11.38 (10.54–12.27)	5.92 (5.33–6.57)	6.88 (6.24–7.57)	2.90 (2.49–3.35)	
Female^b^	n/N (%)	150/546 (28%)	125/510 (25%)	169/672 (25%)	82/359 (23%)	139/426 (33%)	60/184 (33%)	725/2697 (27%)
Children^c^	n/N (%)	197/465 (42%)	167/439 (38%)	198/589 (34%)	96/336 (29%)	155/383 (41%)	78/169 (46%)	891/2381 (37%)
Vehicle Occupant^a^	N (%)	205 (27%)	106 (17%)	36 (5%)	37 (9%)	131 (23%)	219 (54%)	734 (21%)
	Rate per 100,000 persons (CI)	3.63 (3.15–4.16)	1.83 (1.50–2.22)	0.61 (0.43–0.82)	0.61 (0.43–0.82)	2.11 (1.77–2.51)	3.45 (3.01–3.94)	
Female^b^	n/N (%)	12/160 (8%)	9/103 (9%)	4/36 (11%)	10/34 (29%)	3/131 (2%)	34/216 (16%)	72/680 (11%)
Children^c^	n/N (%)	9/182 (5%)	5/101 (5%)	4/32 (13%)	5/36 (14%)	5/118 (4%)	29/196 (15%)	57/665 (9%)
Total	N	751	617	710	396	558	403	3435
	Rate per 100,000 persons (CI)	13.29 (12.35–14.27)	10.67 (9.84–11.54)	11.99 (11.12–12.90)	6.53 (5.91–7.21)	8.99 (8.26–9.77)	6.35 (5.74–7.00)	

^a^ The number and rate presented are that of the specified sub category among all traffic accident deaths irrespective of documented age or sex data. Cell percentages represent the percent of traffic accident deaths for the given year (for annual columns) or for all years, 2010 through 2015 (for the total column)

^b^The number and percentage of females among all traffic accident deaths from the specified sub category. Cell percentages represent the percent of females among the sub category deaths for the given year (for annual columns) or for all years, 2010 through 2015 (for the total column). Figures exclude deaths without documented sex

^c^ The number and percentage of children (persons under 18 years of age) among all traffic accident deaths from the specified sub category. Cell percentages represent the percent of children among the sub category deaths for the given year (for annual columns) or for all years, 2010 through 2015 (for the total column). Figures exclude deaths without documented age

**Table 4:** Annual Other Unintentional Injury Deaths by Injury Type, Sex and Age — Baghdad, Iraq, 2010–2015

Year		2010	2011	2012	2013	2014	2015	Total
Burns^a^	N (%)	377 (48%)	379 (57%)	454 (51%)	472 (42%)	328 (46%)	375 (40%)	2385 (46%)
	Rate per 100,000 persons (CI)	6.67 (6.01–7.38)	6.55 (5.91–7.25)	7.67 (6.98–8.41)	7.79 (7.10–8.52)	5.29 (4.73–5.89)	5.90 (5.32–6.53)	
Female^b^	n/N (%)	229/376 (61%)	257/379 (68%)	335/454 (74%)	337/472 (71%)	235/328 (72%)	281/374 (75%)	1674/2383 (70%)
Children^c^	n/N (%)	102/334 (31%)	89/350 (25%)	104/411 (25%)	106/424 (25%)	111/301 (37%)	87/350 (25%)	599/2170 (28%)
Electrical^a^	N (%)	299 (38%)	215 (32%)	269 (30%)	328 (29%)	230 (32%)	272 (29%)	1613 (31%)
	Rate per 100,000 persons (CI)	5.29 (4.71–5.93)	3.72 (3.24–4.25)	4.54 (4.02–5.12)	5.41 (4.84–6.03)	3.71 (3.24–4.22)	4.28 (3.79–4.82)	
Female^b^	n/N (%)	67/299 (22%)	54/215 (25%)	57/269 (21%)	78/328 (24%)	60/230 (26%)	76/272 (28%)	392/1613 (24%)
Children^c^	n/N (%)	100/263 (38%)	63/199 (32%)	91/242 (38%)	134/331 (41%)	80/222 (36%)	101/259 (39%)	569/1516 (38%)
Drowning^a^	N (%)	66 (8%)	58 (9%)	75 (8%)	76 (7%)	85 (12%)	66 (7%)	426 (8%)
	Rate per 100,000 persons (CI)	1.17 (0.90–1.47)	1.00 (0.76–1.28)	1.27 (1.00–1.57)	1.25 (0.99–1.55)	1.37 (1.09–1.68)	1.04 (0.80–1.30)	
Female^b^	n/N (%)	10/66 (15%)	9/58 (16%)	11/75 (15%)	15/76 (20%)	11/85 (13%)	13/66 (20%)	69/426 (16%)
Children^c^	n/N (%)	35/48 (73%)	20/50 (40%)	41/66 (62%)	42/66 (64%)	53/80 (66%)	38/64 (59%)	229/374 (61%)
Blunt^a^	N (%)	8 (1%)	16 (2%)	55 (6%)	70 (6%)	5 (1%)	120 (13%)	274 (5%)
	Rate per 100,000 persons (CI)	0.14 (0.06–0.26)	0.28 (0.16–0.43)	0.93 (0.70–1.19)	1.15 (0.90–1.44)	0.08 (0.03–0.17)	1.89 (1.57–2.26)	
Female^b^	n/N (%)	2/8 (25%)	7/16 (44%)	18/55 (33%)	17/70 (24%)	4/5 (80%)	34/120 (28%)	82/274 (30%)
Children^c^	n/N (%)	4/4 (100%)	13/14 (93%)	29/49 (59%)	41/60 (68%)	4/5 (80%)	42/107 (39%)	113/239 (47%)
Other^a^	N (%)	43 (5%)	13 (2%)	41 (5%)	184 (16%)	62 (9%)	109 (12%)	452 (9%)
	Rate per 100,000 persons (CI)	0.76 (0.58–1.05)	0.22 (0.12–0.36)	0.69 (0.50–0.92)	3.04 (2.61–3.51)	1.00 (0.77–1.26)	.72 (1.41–2.07)	
Female^b^	n/N (%)	16/43 (37%)	5/13 (39%)	14/40 (35%)	59/184 (32%)	16/62 (26%)	34/109 (31%)	144/451 (32%)
Children^c^	n/N (%)	34/39 (87%)	6/9 (67%)	18/35 (51%)	58/170 (34%)	29/57 (51%)	46/102 (45%)	191/412 (46%)
Total^a^	N	793 (28%)	681 (29%)	894 (33%)	1130 (34%)	710 (21%)	942 (32%)	5150 (29%)
	Rate per 100,000 persons (CI)	14.03 (13.07–15.04)	11.77 (10.90–12.69)	15.10 (14.12–16.12)	18.64 (17.57–19.76)	11.44 (10.62–12.32)	14.83 (13.90–15.81)	

^a^ The number and rate presented are that of the specified sub category among all unintentional deaths irrespective of documented age or sex data. Cell percentages represent the percent of unintentional deaths for the given year (for annual columns) or for all years, 2010 through 2015 (for the total column)

^b^The number and percentage of females among all unintentional deaths from the specified sub category. Cell percentages represent the percent of females among the sub category deaths for the given year (for annual columns) or for all years, 2010 through 2015 (for the total column). Figures exclude deaths without documented sex

^c^ The number and percentage of children (persons under 18 years of age) among all unintentional deaths from the specified sub category. Cell percentages represent the percent of children among the sub category deaths for the given year (for annual columns) or for all years, 2010 through 2015 (for the total column). Figures exclude deaths without documented age

**Fig. 2:**
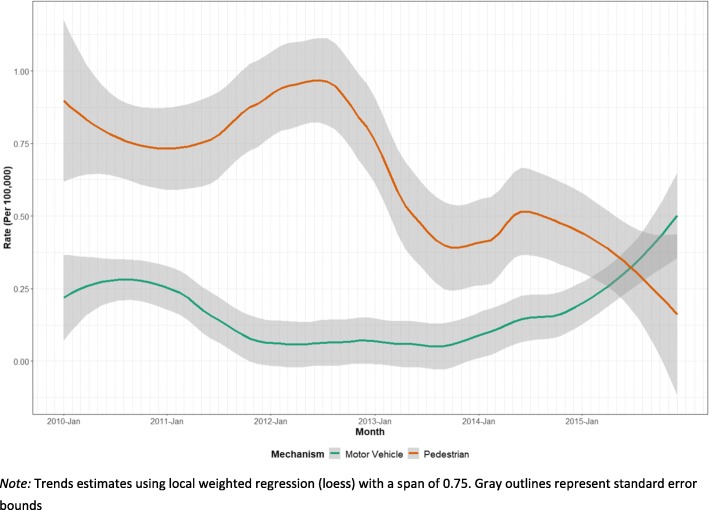
Traffic Accident Deaths by Month — Baghdad, Iraq, 2010–2015 (*N* = 3381)

Pedestrian deaths made up the majority of traffic accident deaths (79%) (Table 3, Fig. 2). The decrease in traffic accident deaths during the study period was primarily attributable to a 70% decline in the rate of pedestrian deaths between 2010 and 2015 (*p* < 0.001). In 2010, pedestrian deaths made up 73% of traffic accident deaths, however, in 2015 vehicle occupant deaths outnumbered pedestrian deaths for the first time. The proportion of female and child deaths was higher among pedestrian deaths (27% female, 37% children) than vehicle occupant deaths (11% female, 9% children); these proportions were relatively unchanged throughout the study period.

**Fig. 3:**
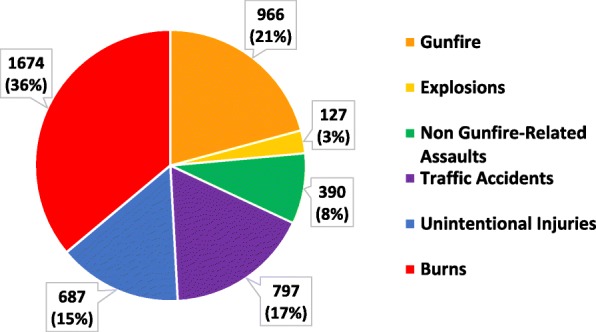
Injury Deaths in Females by Category — Baghdad, Iraq, 2010–2015 (*N* = 4641)

Other unintentional injury deaths were primarily due to burns (46%) and electrical injuries (31%) (Table 4). Electrical injury deaths were primarily among males (76%) whereas unintentional burn deaths were primarily among females (70%). Overall, 36% of all female injury deaths were from unintentional burns (Fig. 3), outnumbering female deaths from gunfire, explosions and non-gunfire-assault combined (32%). Around half of unintentional drownings deaths (61%) and unintentional blunt injury deaths (47%) were in children.

## Discussion

Registry data demonstrates that the number and rate of deaths from insurgency-related injuries in Baghdad were relatively stable from 2010 to 2012, but increased in 2013 and 2014. This increase coincided with the emergence of the Islamic State. This trend is consistent with that documented by other sources, both in Baghdad and Iraq nationally, which demonstrated relatively low numbers of conflict deaths from 2010 through 2012 with a subsequent increase in mortality in 2013 [2–11]. Data from the Global Burden of Disease study suggest that Iraq was second only to Syria in 2014 in terms of overall conflict mortality [15, 24, 25]. Furthermore, in both 2014 and 2015, Baghdad experienced the most terror attacks of any city worldwide, suggesting that Baghdad remained the city most affected by terror attacks even following the observed decline in conflict-fatalities in 2015 [25].

Research by Tessler et al. has shown that while explosive devices may be the most common method used in terror attacks worldwide, gunfire accounts for 55% of total terrorism-related mortality in high-income countries [26]. This registry data documents four times higher injury mortality from gunfire despite evidence that the majority of terrorism-related incidents in Bagdad utilized explosives. According to the Global Terror Database, 87% of total terror attacks in Baghdad from 2010 to 2015 utilized explosives and only 12% used gunfire [25]. Our findings of higher gunfire deaths may be due to several factors. One possibility is that gunfire-based terror attacks in Baghdad may have higher associated mortality compared to those involving explosives. This is supported by a prior study of terror attacks from 1968 to 2004 that demonstrated the case fatality for gunfire attacks to be 2.5 times higher than that of explosions [27]. Alternatively, gunfire use may be an underreported form of terrorism in Baghdad as media reports predominately focus on attacks involving explosive devices [28].

Non-gunfire-related assault deaths increased steadily starting in 2011, almost doubling from 2011 to 2015. This trend continued despite relative decreases in conflict deaths in 2015. While some studies have demonstrated similar increases in non-conflict violence in other countries during periods of conflict and post-conflict recovery, the impact of conflict on homicide and other crime is nuanced, and likely impacted by many factors such as historical context and relations between religious or ethnic groups [29, 30]. An increase in domestic violence associated with conflict has been observed in other settings; research in Colombia reported that women living in rural areas most affected by conflict were more likely to have experienced domestic violence in the past year compared to those in less conflict affected areas [31]. Our surveillance data demonstrated that non-gunfire-assault deaths in females doubled from 2010 to 2015 and tripled amongst children in Baghdad. The proportion of females and children was highest in suffocation deaths, a common method of domestic abuse and a high predictor of future homicide in non-fatal cases [32]. Data on suffocations are limited in low to medium income countries; however, a multiple country evaluation reported that national past-year prevalence of non-fatal strangulation in women ranged from 0.4 to 2.4% [33].

Traffic accident deaths decreased overall from 2010 to 2015, a decline driven primarily by reductions in fatalities amongst pedestrians. This may be due both to decreased road activity during the ongoing conflict and to specific interventions to reduce traffic mortality [34]. Iraq has called for a “Decade of Action for Road Safety” from 2010 to 2020, which could also explain the relative decrease in traffic accident deaths. However, in 2014, the rate of vehicle occupant deaths (a subgroup of traffic accident deaths) began to increase and continued to their highest level in 2015 (see Fig. 2). Iraq has limited traffic laws, and those that exist are irregularly enforced [34]. WHO reports further note that Iraq has limited regular inspections of existing road infrastructure and no policies on child restraint laws, vehicle impact standards, or separation of other road users from vehicles (e.g., bikes, pedestrians). Thus, additional policies aimed at reducing vehicle occupant deaths may slow this rise in mortality as people return to the roads during the post-conflict period [35].

Other unintentional injury deaths were primarily due to burns. Representing nearly 10% of injury deaths among both sexes, unintentional burns were a primary mechanism of injury mortality overall in Baghdad. Notably, unintentional burn deaths occurred primarily among females (70%) and accounted for 36% of all female injury deaths. Burns are a common cause of death in women in the Middle East region and have been reported to be from a combination of household exposures, interpersonal violence, and suicide [36]. However, few female burn deaths in this analysis were classified as intentionally inflicted (2%) and none were classified as self-harm. This contrasts with other findings in Iraq. Reports have shown burns are a common mechanism of domestic violence in Iraq [37, 38]. In addition, prospective surveillance from a Burn and Plastic Surgery Hospital in Kurdistan showed that 83% of burn deaths in women were suicides [39]. Given existing research on the use of burning as a common mechanism for domestic violence and self-harm in the region, it is possible that fatal burns classified in our data as unintentional may have been attributable to either interpersonal violence or self-harm, resulting in an underestimation of deaths from these categories.

The findings presented here are subject to several limitations. First, Iraq has not had a national census since 1997. As a result, updated census figures for Baghdad governorate are not available. Modeled estimates from the World Bank may underestimate the total population under surveillance, as they (1) are for Baghdad metropolitan area which does not include sub-urban and peri-urban areas of Baghdad governorate covered by the surveillance system; and (2) do not account for temporary displacement due to violence [19, 40]. Reported trends in fatalities likely represent both changes in risk as well as changes in the population at risk. Second, the small number of deaths amongst certain subcategories created unstable annual trends. Third, while the proportion of fatalities missing sex and cause of death was low (< 2%), a large number of deaths had undocumented age (17%). Revision of the surveillance forms to allow for estimation of age when exact date of birth is unknown would improve future reporting. The proportion of fatalities missing age (as well as other variables) was reasonably consistent throughout the study period providing some evidence that factors such as increased conflict violence did not meaningfully impact quality of reporting*.* Fourth, collected data does not link individual deaths to a specific event or attack, making it difficult to ascertain how many individual explosions or gunfire attacks led to observed deaths. Fifth, both self-harm and domestic violence deaths may be under-reported, as evidenced by a higher than expected proportion of fatal burns in women attributed to unintentional injuries and no domestic violence reported in some years. Other studies have demonstrated high numbers of domestic violence deaths in other areas of Iraq and increasing rates of suicide in both sexes [39, 41]. Domestic violence in Iraq is also considered honorable in some situations, and both mental health and self-harm carry stigma, factors that may impact both reporting and classification [42–45].

Finally, surveillance systems, particularly those in low- and middle-income countries, generally suffer from imperfect sensitivity; as such, it is possible that deaths occurring in Baghdad during the study period were missed [46, 47]. While there has not been a formal evaluation of the surveillance system’s sensitivity, previous independent researchers have reported that throughout periods of greatest insecurity in Iraq, issuance of death certificates remained nearly universal [9–11]. However, there remains the possibility that deaths with issued death certificates may not be registered in the surveillance system. Changes in sensitivity following the insurgency, if present, could impact interpretation of trends. While such changes are possible, we note that quality of reporting (as indicated by the proportion of fatalities with incomplete data) was relatively stable over the six-year period included in our study. Additionally, annual trends reported here are consistent with those reported by other researchers using different epidemiologic methods (i.e., cross-sectional household surveys) [6, 9–11]. Also, specific injury cause categories could have differing sensitivities, such as the discussed concern regarding domestic violence and suicides. If such differences exist, this could affect our proportionate mortality estimates. However, several of our findings are similar to those from aforementioned population-representative surveys in Baghdad City conducted by independent researchers (e.g., gunfire caused a larger proportion of deaths than explosions as well as similar proportions of deaths from explosions and non-gunfire-assault). While the evidence mentioned in this paragraph provides reassurance about system’s internal validity as well as reliability of presented trends and proportional mortality causes, completeness of reporting remains a serious concern, and available data does not allow for providing an estimate of what percentage of injury deaths remain unreported. Ultimately, given the system has been operational for nearly a decade, a comprehensive and systematic evaluation of sensitivity and other key quality parameters of the system is needed.

## Conclusions

Deaths from both gunfire and explosions in Baghdad peaked during the study period in 2014, corresponding with emergence of the Islamic State. Insurgency-related activity is associated with an increase in deaths from non-gunfire-assault and a decrease in deaths from traffic accidents. Increases in non-gunfire-related assault deaths could be due to increases in crime or misclassification of domestic violence. Decreased traffic-related deaths could be from decreased vehicle and pedestrian activity during times of violence. Unintentional injury deaths varied throughout the study period though most of these deaths were from burns in women.

Surveillance data is critical to public health planning and policy, and remains vital during crises in order to inform humanitarian response and allocation of resources. This analysis provides information on trends in both conflict- and non-conflict-related injury mortality in Iraq and explores how data can be used to guide immediate public health interventions. These findings demonstrate similar proportionate mortality to other conflicts and can potentially be utilized in future crises to anticipate the needs of certain populations. Conflict settings with prospective injury surveillance remain limited. Therefore, additional attention and resources concentrated in conflict-affected areas to provide injury surveillance information would enable the restoration, maintenance, and advancement of public health.

## Data Availability

The authors confirm that, for approved reasons, some access restrictions apply to the data underlying the findings. Although the subject-level data do not include names, this decision is in the interest of ensuring confidentiality of subjects and their families. Data can be made available on request by contacting the lead author (nqy3@cdc.gov) or the Ministry of Health Iraq (ahmedhr_1969@yahoo.com).

## Abbreviations

CDC: Centers for Disease Control and Prevention
ISIL: Islamic State of Iraq and the Levant
ISIS: Islamic State of Iraq and Syria
SAS: Statistical Analysis System
UN: United Nations
US: United States
WHO: World Health Organization
