# Correction to: Injury-related deaths before and during the Islamic State insurgency – Baghdad, Iraq, 2010–2015

**DOI:** 10.1186/s13031-020-00261-5

**Published:** 2020-03-04

**Authors:** Matthew Goers, Eva Leidman, Abdul-Salam Saleh Sultan, Ahmed Hassan, Oleg Bilukha

**Affiliations:** 1 Centers for Disease Control and Prevention, Center for Global Health, Division of Global Health Protection, Atlanta, GA USA; 2 National Training and Human Development Center, Iraq Ministry of Health, Bab Al Mu’adham Street, Baghdad, Iraq; 3 Medical Operations and Specialized Services Directorate, Operations Department, Iraq Ministry of Health, Al Adham Street, Baghdad, Iraq

**Correction to: Confl Health (2020) 14:8**

**https://doi.org/10.1186/s13031-020-0252-7**

The original publication of this article [1] contained an incorrect author name. The correct and incorrect information is shown in this correction article. The original article has been updated.

The incorrect author name was published as:

Abdul-Salam Saleh Sultran

The correct author name is:

Abdul-Salam Saleh Sultan
